# ZNF143 facilitates the growth and migration of glioma cells by regulating KPNA2-mediated Hippo signalling

**DOI:** 10.1038/s41598-023-38158-x

**Published:** 2023-07-09

**Authors:** Yan Chen, Jitao Li, Jiangchun Ma, Yizhong Bao

**Affiliations:** 1 Department of Neurosurgery, The Second Hospital of Zhejiang University School of Medicine, Hangzhou, 310009 People’s Republic of China; 2 Zhejiang Provincial Key Lab of Geriatrics, Department of Geriatrics, Zhejiang Hospital, Hangzhou, 310013 People’s Republic of China; 3 Department of Neurosurgery, Zhejiang Hospital, Hangzhou, 310013 People’s Republic of China; 4 Department of Oncology, Shengli Oilfield Central Hospital, Dongying, 257034 People’s Republic of China

**Keywords:** Cancer, Cell biology, Neuroscience

## Abstract

The disordered expression of ZNF143 is closely related to the malignant progression of tumours. However, the basic control mechanism of ZNF143 in glioma has not yet been clarified. Therefore, we tried to find a new pathway to illustrate the function of ZNF143 in glioma. To explore the function of KPNA2 in the development of glioma, we used survival analysis by the Kaplan‒Meier method to assess the overall survival (OS) of patients with low and high KPNA2 expression in the TCGA and CGGA cohorts. Western blotting assays and RT‒PCR assays were utilized to determine the expression level of KPNA2 in glioma cells. The interaction between ZNF143 and KPNA2 was confirmed by ChIP assays. Proliferation was assessed by CCK-8 assays, and migration was evaluated by wound healing and Transwell assays. Apoptosis was determined by flow cytometry, and the expression level of YAP/TAZ was visualized using an immunofluorescence assay. The expression levels of LATS1, LATS2, YAP1, and p-YAP1 were determined. Patients with low KPNA2 expression showed a better prognosis than those with high KPNA2 expression. KPNA2 was found to be upregulated in human glioma cells. ZNF143 can bind to the promoter region of KPNA2. Downregulation of ZNF143 and KPNA2 can activate the Hippo signalling pathway and reduce YAP/TAZ expression in human glioma cells, thus inducing apoptosis of human glioma cells and weakening their proliferation, migration and invasion. In conclusion, ZNF143 mediates the Hippo/YAP signalling pathway and inhibits the growth and migration of glioma cells by regulating KPNA2.

## Introduction

Glioma is the most common fatal malignant primary brain tumour and is highly invasive with a poor prognosis. Currently, considerable progress has been achieved in the treatment of glioma. However, the median survival time of glioma patients is only 14.5–16.6 months^1^. The pathological mechanisms of gliomas are quite complex and play a crucial role in tumour development^2^. Therefore, a deeper understanding of the molecular mechanisms of glioma is important for the discovery of prognostic biomarkers and therapeutic targets.

The Hippo signalling pathway is a highly conserved pathway among different organisms, as well as in different tissues and organs in an individual organism, and regulates a variety of biological functions of malignant tumours, including proliferation and apoptosis. Hippo signalling plays a critical role in the pathogenesis and development of tumours^3^. Moreover, the Hippo signalling pathway is involved in the regulation of cell proliferation and apoptosis, control of organ size, and maintenance of the internal tissue environment. Dysregulation of Hippo signalling is observed in multiple types of malignant tumours and is closely associated with the pathogenesis of malignant tumours^4^. The phosphorylation of LATS1/2 is induced by the activation of Hippo signalling, which further represses the activity of transcriptional coactivators, such as YAP and TAZ^5^. Karyopherin alpha 2 (KPNA2) is one of seven known nuclear transporter α proteins that play an important role in nuclear cytoplasmic transport^6^. Dysfunctions in nucleocytoplasmic transport are commonly observed in malignant tumours, and KPNA2 is highly expressed in a variety of malignant tumours. These findings suggest that KPNA2 is possibly involved in the occurrence and development of a variety of tumours^7^. However, the exact mechanism of KPNA2 in gliomas remains unclear. The present study aims to investigate the role of ZNF143 (zinc finger protein 143, the transcription factor of KPNA2) in regulating the effects of KPNA2 on glioma and to explore novel therapeutic targets for glioma.

## Materials and methods

### Data collection and data processing

Data of TPM expression values that contain 33 TCGA pan-cancer and GTEX RNA Seq data were downloaded from the TOIL project via the UCSC Xena Browser. The Chinese Glioma Genome information and the relevant clinical resources were downloaded from the CGGA (Chinese Glioma Genome Atlas) data portal.

### Survival analysis

Survival analysis by the Kaplan‒Meier method was used to assess the OS of patients in the low- and high-expression groups of KPNA2 in the TCGA and CGGA cohorts. The log-rank test was used to assess statistical significance using the R package “survival” and “survminer”. A *P* value < 0.05 was considered statistically significant.

### Cells and treatments

U373 cells (BNCC338603, BNCC, China) and NHA cells (BFN60808805, BLUEFBIO, China) were cultured in DMEM with 10% FBS under 5% CO_2_ and 37 ℃.

### Real-time PCR

In brief, RNA was extracted utilizing TRIzol reagent and further transcribed into cDNA with HiScript II Q RT SuperMix for qPCR (+ gDNA wiper) (R223-01, Vazyme, Nanjing, China). PCR was conducted using 2 × SYBR Green PCR Master Mix (A4004M, Lifeint, China), followed by determining the expression of genes with the 2^−ΔΔCt^ method after normalization to the expression of β-actin. The sequences of the primers are listed in Table 1.

**Table 1:** Sequences of primers in the RT-PCR assay.

Genes	Sequences (5′-3′)
KPNA2 F	TGAGGCGTCGCAGAATAGA
KPNA2 R	GGAGAAGTAGCATCATCAGGAAAT
ZNF143 F	CCCATACCTAAAAGTACAGGGGA
ZNF143 R	TCATTCCAGTACCTGCTACACTT
β-actin F	TGGCACCCAGCACAATGAA
β-actin R	TGGCACCCAGCACAATGAA

### Western blotting assay

Cells were lysed with cell lysate for 30 min. The lysate was transferred to a 1.5 mL centrifuge tube for centrifugation at 12,000 r/min at 4 ℃ for 5 min. The total protein concentration of the supernatant was determined by the BCA method. After 15% SDS‒PAGE electrophoresis separation, proteins were transferred to PVDF membranes, which were sealed with 5% skim milk powder and shaken at room temperature for 1.5 h. The PVDF membrane was removed and rinsed with TBST solution 3 times and placed in a clean dish. LAST1 (1:500, DF7517, Affinity, Australia), LAST2 (1:500, DF7516, Affinity, Australia), YAP1 (1:500, 13584-1-ap, Proteintech, USA), p-YAP1 (1:500, AF3328, Affinity, Australia), KPNA2 (1:500, 10819-1-ap, Proteintech, USA), ZNF143 (1:500, 16618-1-ap, Proteintech, USA), β-actin (1:2000, TA-09, SolelyBio, China) and GAPDH (1:2000, TA-08, SolelyBio, China) primary antibodies were added overnight at 4 °C. Before hybridization, the PVDF membrane was cropped according to the molecular weight of the proteins to be detected. TBST solution was added to rinse 3 times, and the secondary antibody (1:2000, ZB-2305, SolelyBio, China) was added and incubated at room temperature for 1.5 h. The PVDF membrane was removed and rinsed with TBST solution 3 times, and bands were obtained after exposure to ECL solution, which were further quantified using ImageJ software.

### Establishment of KPNA2 knockdown and ZNF143 knockdown glioma cells

Three siRNAs targeting KPNA2 and ZNF143 were constructed, the sequences of which are shown in Table 2. Glioma cells were transfected with 10 μL of siRNA together with 5 μL Lipofectamine 3000 (L3000015, Invitrogen, USA) for 48 h, followed by evaluation of the transfection efficacy using Western blotting and RT‒PCR assays.

**Table 2:** Sequences of siRNAs.

siRNAs	Sequences (5′-3′)
KPNA2-siRNA-1	GCUGCCAGGAAACUACUUUTT AAAGUAGUUUCCUGGCAGCTT
KPNA2-siRNA-2	GCAGAUUCUUCCUACCUUATT UAAGGUAGGAAGAAUCUGCTT
KPNA2-siRNA-3	GCUGAGAAACUAGGUGAAATT UUUCACCUAGUUUCUCAGCTT
ZNF143-siRNA-1	AUAUCGGUGUUCGGAAGAUAATT UUAUCUUCCGAACACCGAUAUTT
ZNF143-siRNA-2	GCGGCCUAUGUUCAACAUGUATT UACAUGUUGAACAUAGGCCGCTT
ZNF143-siRNA-3	CGCACUCUGUUGCUAUGGUUATT UAACCAUAGCAACAGAGUGCGTT
siRNA-NC	UUCUCCGAACGUGUCACGUTT ACGUGACACGUUCGGAGAATT

### Cell viability

A CCK-8 assay was utilized to evaluate cell viability. Cells were plated in 96-well plates and incubated at 37 °C for 24 h. Subsequently, 10 μL of CCK-8 solution was added, and the absorption was measured at 450 nm with a microplate reader (S/N502000011, Tecan, Switzerland).

### Analysis of apoptosis

The apoptosis of treated cells was evaluated using flow cytometry. In brief, cells were plated on 6-well plates and incubated with 195 µL of Annexin V-fluorescein isothiocyanate (AP101-100-kit, Multi Sciences, China), followed by the addition of 5 µL of propidium iodide and incubation for 10 min at room temperature in the dark for 10 min. Finally, the samples were loaded onto the flow cytometer (NovoCyte 2060R, ACEABiosciences, USA) for apoptosis analysis.

### Transwell assay

In brief, cells were collected, counted, and seeded into a Transwell insert (3422, Corning, New York, USA) at a density of 1.5 × 10^5^ cells per well for the migration assay. The lower chamber was filled with medium containing 20% FBS. Cells were then cultured in serum-free medium in the upper chamber for 24 h at 37 °C and 5% CO_2_, followed by wiping off cells from the upper chambers. Subsequently, cells from the lower chambers were stained with crystal violet and counted under an optical microscope (CX41, Olympus, Japan).

### Wound healing assay

A 200 μL pipette tip was used to scratch and draw a line in each well, and the medium was discarded. The culture medium was cleaned with PBS 3 times, and a picture of the scratch in each well was taken (0 h) after the serum-free culture medium was replaced. Cells were placed in an incubator, and the scratches of each well were photographed 48 h later (48 h). The width of the scratch at 0 h and 48 h was measured, and then the migration rate of cells was calculated.

### Immunofluorescence

Cells were immersed in PBS 3 times and fixed with 4% paraformaldehyde. Triton X-100 (0.5%) was permeated at room temperature for 20 min, followed by the addition of 5% BSA at 37 °C for 30 min. The primary antibody against YAP/TAZ (1:150, D24E4, CST, USA) was then added and incubated at 37 °C for 3 h. A fluorescent secondary antibody (1:200, AS007, ABclonal, China) was added and incubated at 37 °C for 30 min in the dark. DAPI was then added, followed by incubation at 37 °C in the dark for 5 min to stain the nucleus. The petri dish was sealed with 50% glycerol, and then the images were observed and collected under a fluorescence microscope (CKX53, Olympus, Japan).

### ChIP

Since ZNF143 is a transcription factor of KPNA2, to confirm the interaction between ZNF143 and KPNA2, we generated a KPNA2 overexpression vector. After transfection, Western blotting and RT‒PCR assays were used to verify the transfection efficiency. Subsequently, ZNF143 antibody was added and combined with the target protein‒DNA complex to form the antibody-target protein‒DNA complex. After elution of the precipitated complex, the enriched target protein‒DNA complex was obtained, and the enriched DNA fragment was purified. The interaction between KPNA2 and ZNF143 was detected by RT‒PCR.

### Statistical analysis

Data are expressed as the mean ± SD, and data analysis was conducted using GraphPad software. One-way ANOVA was used for the analysis among groups using Tukey’s method. *P < *0.05 was considered a significant difference.

## Results

### The mRNA expression of KPNA2 is increased in multiple malignant tumours

**Figure 1:**
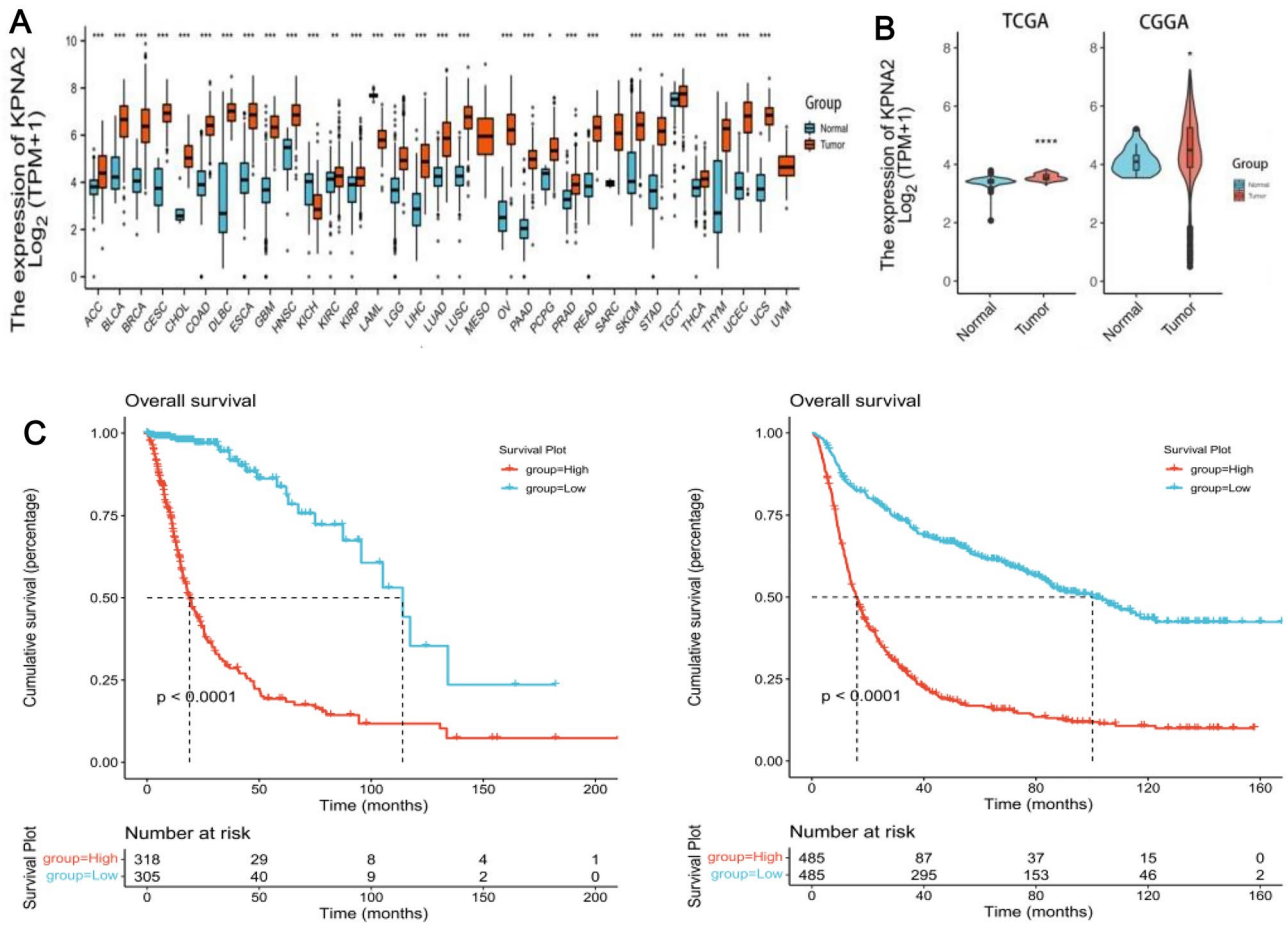
Patients with low KPNA2 expression showed a better prognosis than those with high KPNA2 expression in both the TCGA and CGGA databases. KPNA2 was expressed in 33 types of malignant tumours compared to normal tissues (**A**); KPNA2 was expressed in glioma either in the TCGA database or CGGA database (**B**); Prognostic value of KPNA2 in glioma in the TCGA database or CGGA database (**C**).

Our analyses showed that KPNA2 expression was increased in 28 types of malignant tumors, the full and abbreviated names of which are shown in Table 3, as compared to the normal tissues (Fig. 1A).

**Table 3.** 28 types of malignant tumors.

ACC	Adrenocortical carcinoma
BLCA	Bladder urothelial carcinoma
BRCA	Breast invasive carcinoma
CESC	Cervical squamous cell carcinoma and endocervical adenocarcinoma
CHOL	Cholangiocarcinoma
COAD	Colon adenocarcinoma
DLBC	Lymphoid neoplasm diffuse large B-cell lymphoma
ESCA	Esophageal carcinoma
GBM	Glioblastoma multiforme
HNSC	Head and neck squamous cell carcinoma
KICH	Kidney chromophobe
KIRC	Kidney renal clear cell carcinoma
KIRP	Kidney renal papillary cell carcinoma
LAML	Acute myeloid leukemia
LGG	Brain lower grade glioma
LUSC	Lung squamous cell carcinoma
LIHC	Liver hepatocellular carcinoma
LUAD	Lung adenocarcinoma
MESO	Mesothelioma
OV	Ovarian serous cystadenocarcinoma
PRAD	Prostate adenocarcinoma
PAAD	Pancreatic adenocarcinoma
PCPG	Pheochromocytoma and paraganglioma
READ	Rectum adenocarcinoma
STAD	Stomach adenocarcinoma
SARC	Sarcoma
SKCM	Skin cutaneous melanoma
TGCT	Testicular germ cell tumors
THCA	Thyroid carcinoma
THYM	Thymoma
UCEC	Uterine corpus endometrial carcinoma
UVM	Uveal melanoma
UCS	Uterine carcinosarcoma

Furthermore, we found that KPNA2 was significantly highly expressed in glioma in both the TCGA and CGGA databases (Fig. 1B). These results suggested that KPNA2 was overexpressed in human glioma, including LGG and GBM.

### Prognostic value of KPNA2 in Glioma

After processing raw data from TCGA and CGGA, we separated the glioma data into low and high groups according to KPNA2 expression. Then, we found that patients with low KPNA2 expression had a better prognosis than those with high KPNA2 expression in both the TCGA database and CGGA database (TCGA: n = 623; *P =* 4.535348e-23; HR, 0.12; 95% CI 0.08–0.18, CGGA: n = 970; *P =* 6.984371e-47; HR, 0.29; 95% CI 0.24–0.34, Fig. 1C).

### KPNA2 was highly expressed in human glioma cells

**Figure 2:**
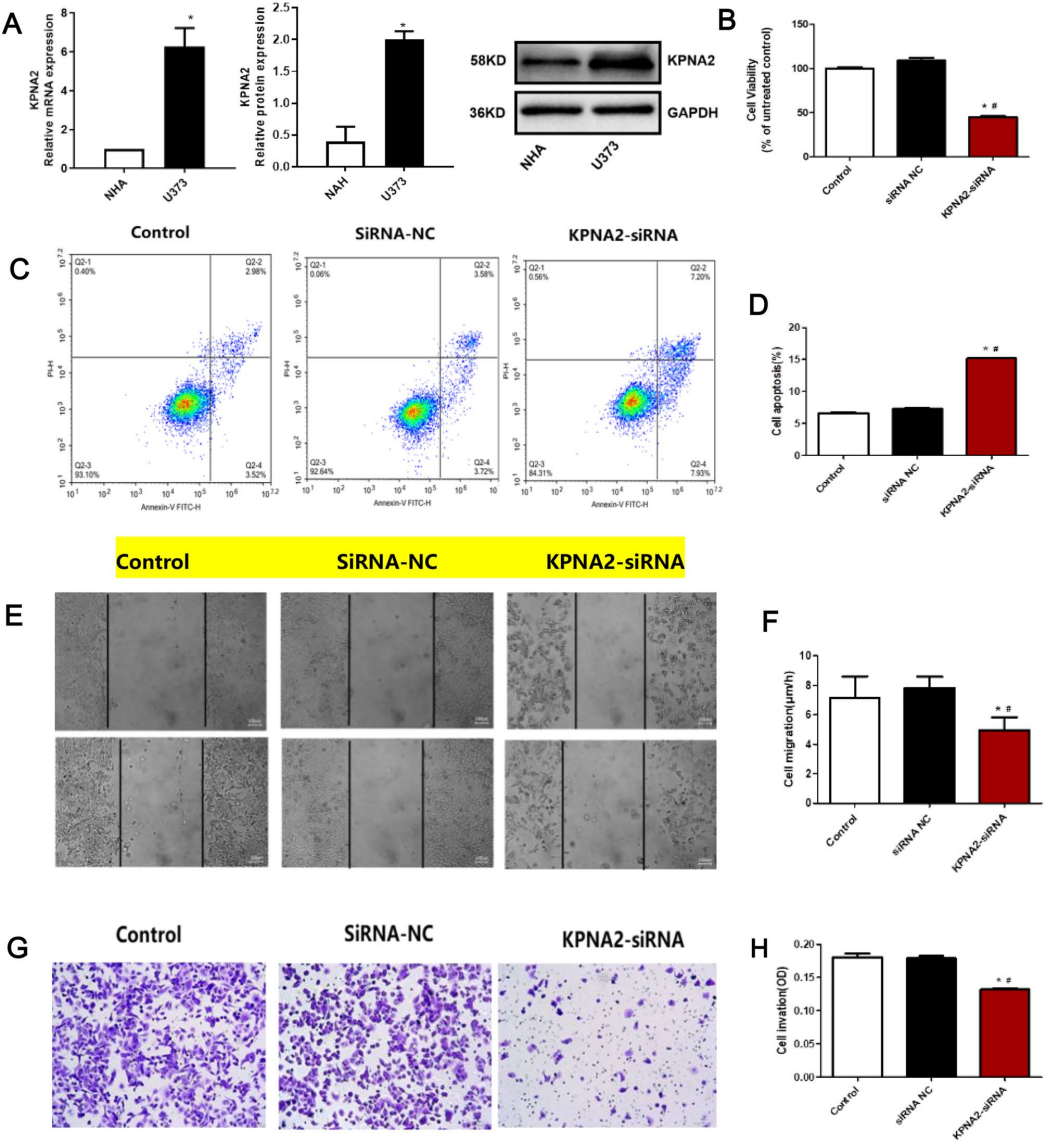
The impact of KPNA2 on the proliferation, migration, and apoptosis of U373 cells. (**A**) The expression level of KPNA2 in U373 and NHA cells was determined by Western blotting and RT‒PCR (*p < 0.05 vs. NHA). (**B**) Cell viability was detected by CCK-8 assays. (**C**, **D**) Apoptosis was evaluated by flow cytometry. (**E**, **F**) The wound healing assay was utilized to determine the migration of U373 cells. (**G**, **H**) The migration of U373 cells was detected using the Transwell assay (*p < 0.05 vs. the control, ^#^ *p < *0.05 vs. siRNA-NC).

As shown in Fig. 2A, compared to NHA cells, KPNA2 was significantly upregulated in U373 cells (*p < 0.05 vs. NHA), which was consistent with the results observed in glioma tissues in the TCGA dataset.

### KPNA2 knockdown U373 cells were successfully established

As shown in Supplementary Fig. 1, compared to those of the control and siRNA-NC groups, the expression level of KPNA2 was dramatically decreased in U373 cells transfected with all three siRNAs, among which the highest knockdown efficacy was observed in U373 cells transfected with KPNA2-siRNA-2 (*p < 0.05 vs. the control, ^#^*p < *0.05 vs. siRNA-NC). Therefore, KPNA2-siRNA-2 was utilized for the knockdown of KPNA2 in U373 cells in subsequent experiments.

### The impact of KPNA2 on the proliferation, migration, and apoptosis of U373 cells

As shown in Fig. 2B, compared to that of the control and siRNA-NC groups, significantly decreased cell viability was observed in the KPNA2-siRNA group, accompanied by a dramatically increased apoptotic rate (Fig. 2C,D). Furthermore, compared to those of the control and siRNA-NC groups, the value of cell migration (Fig. 2E,F) and the number of migrated cells (Fig. 2G,H) were greatly reduced in the U373 cells transfected with KPNA2-siRNA (*p < 0.05 vs. the control, ^#^*p < *0.05 vs. siRNA-NC). These data suggested that the proliferation and migration of U373 cells were dramatically repressed by the knockdown of KPNA2, accompanied by an elevated apoptotic rate.

### YAP/TAZ levels were decreased in KPNA2 knockdown U373 cells

**Figure 3:**
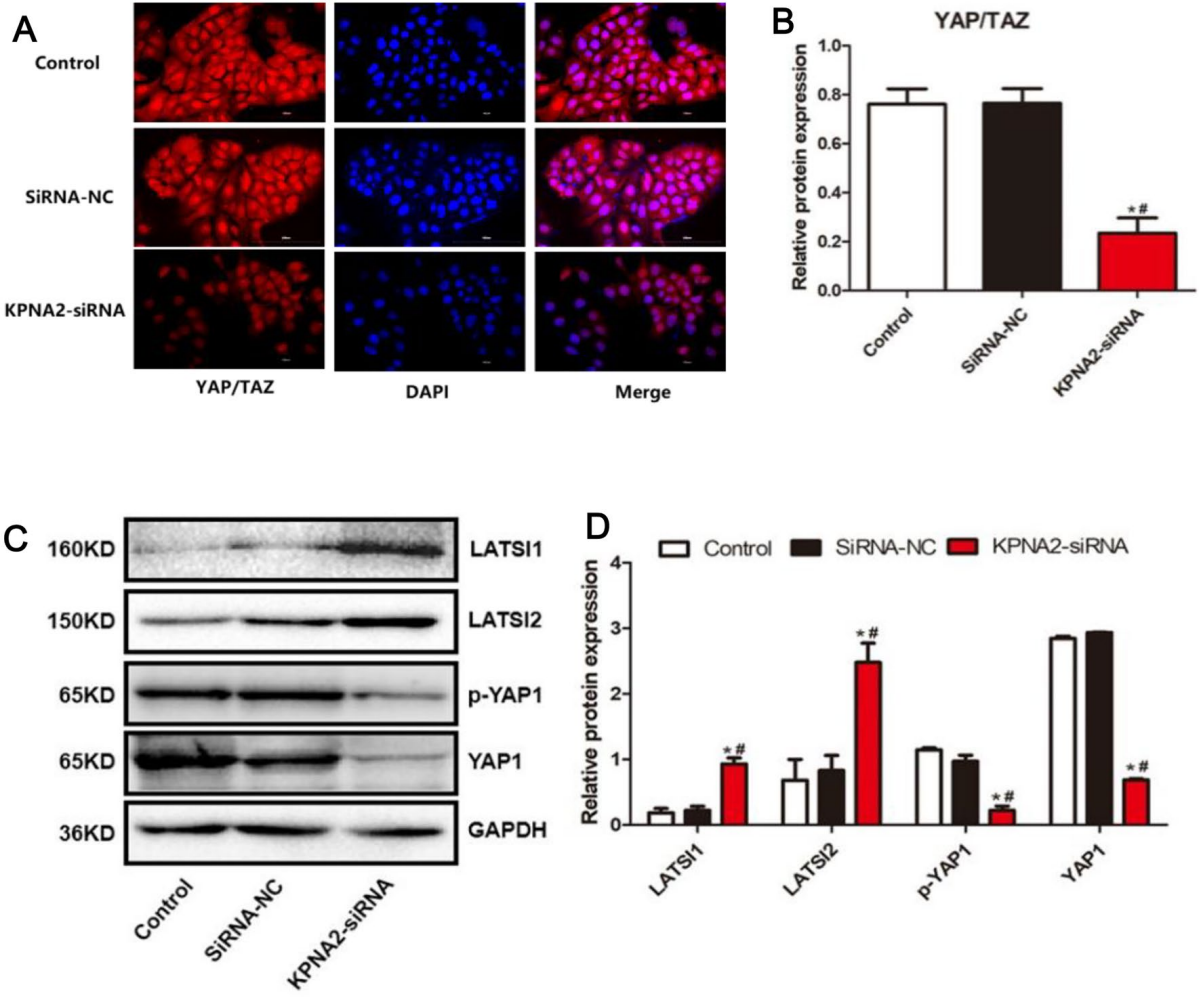
The impact of KPNA2 on the YAP/TAZ pathway. (**A**, **B)** The expression level of YAP/TAZ in U373 cells was determined using immunofluorescence assays (*p < 0.05 vs. the control, ^#^*p < *0.05 vs. siRNA-NC). (**C**, **D**): The expression levels of LATS1, LATS2, YAP1, and p-YAP1 were determined using Western blotting (*p < 0.05 vs. the control, ^#^*p < *0.05 vs. siRNA-NC).

Subsequently, the level of YAP/TAZ was determined using an immunofluorescence assay in KPNA2 knockdown U373 cells. Compared to those of the control and siRNA-NC groups, the fluorescence intensity (Fig. 3A) was dramatically decreased in the U373 cells transfected with KPNA2-siRNA (*p < 0.05 vs. the control, ^#^*p < *0.05 vs. siRNA-NC), suggesting that YAP/TAZ was dramatically downregulated in KPNA2 knockdown U373 cells.

### The impact of KPNA2 on the expression levels of LATS1, LATS2, YAP1, and p-YAP1 in U373 cells

As shown in Fig. 3B, compared to those in the control and siRNA-NC groups, LATS1 and LATS2 were upregulated, while YAP1 and p-YAP1 were downregulated in the U373 cells transfected with KPNA2-siRNA (*p < 0.05 vs. the control, ^#^*p < *0.05 vs. siRNA-NC), suggesting that Hippo signalling in U373 cells was regulated by KPNA2.

### ZNF143 targeted KPNA2 in human glioma cells

**Figure 4:**
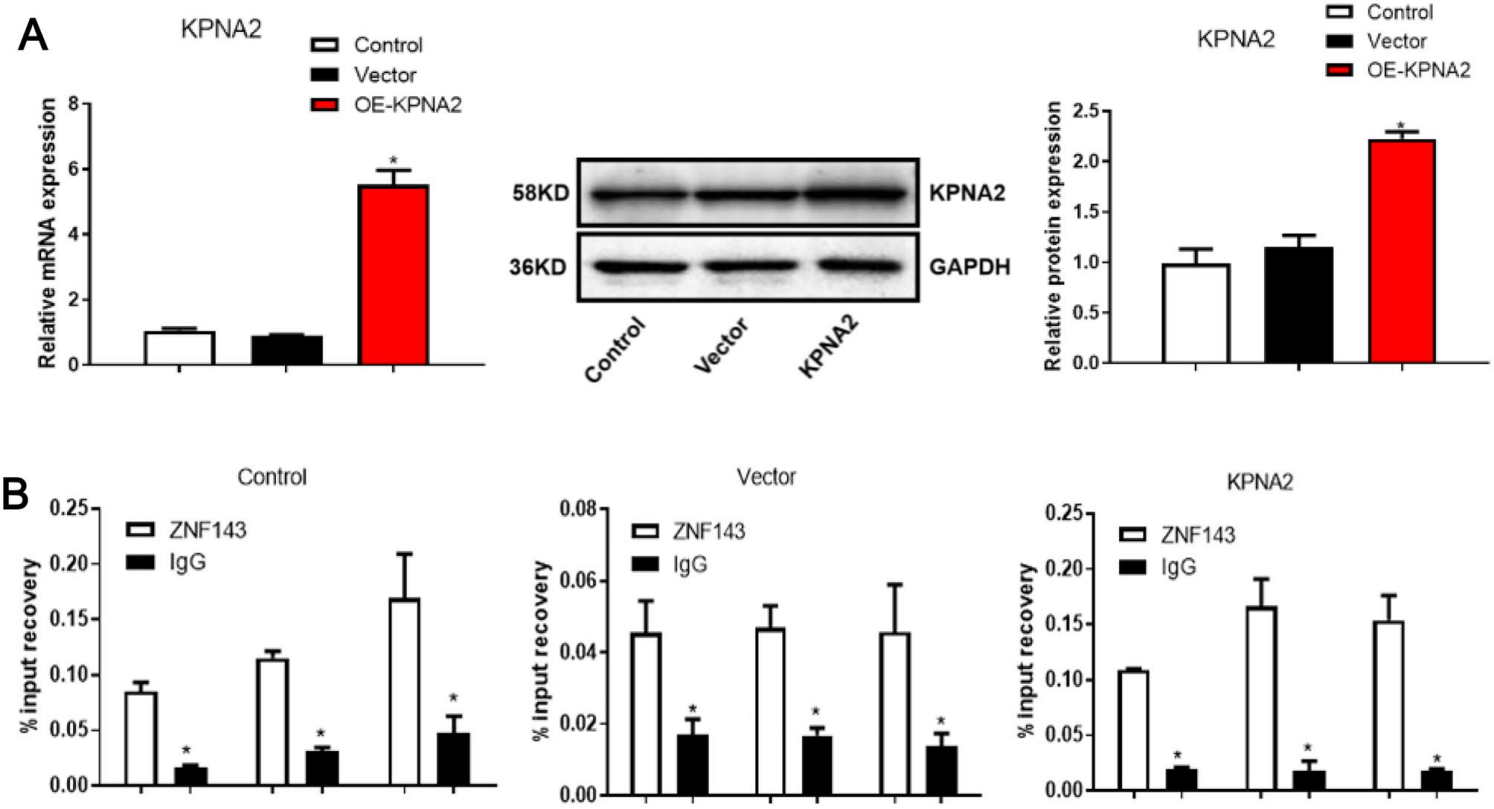
The interaction between ZNF143 and KPNA2 in glioma cells was confirmed using a ChIP assay. (**A**) The identification of the successful transfection of the KPNA2 overexpression vector (*p < 0.05 vs. the control). (**B**) A ChIP assays were utilized to demonstrate the interaction between ZNF143 and KPNA2 in glioma cells.

As shown in Fig. 4, to verify whether there is a binding site between ZNF143 and the promoter regions of KPNA2, we conducted a ChIP assay to detect the interaction between the transcription factor ZNF143 and the promoter of the target gene KPNA2, along with qPCR, which showed that the KPNA2 promoter region was obviously highly expressed in the ZNF143 interaction samples, and the difference was significant compared with that of the IgG group, indicating that the KPNA2 promoter region was bound by ZNF143.

### ZNF143 knockdown U373 cells were successfully established

As shown in Supplementary Fig. 2, compared to those of the control and siRNA-NC groups, the expression level of ZNF143 was dramatically decreased in the U373 cells transfected with all three siRNAs, among which the highest knockdown efficacy was observed in the U373 cells transfected with ZNF143-siRNA-3 (*p < 0.05 vs. the control, ^#^*p < *0.05 vs. siRNA-NC). Therefore, ZNF143-siRNA-3 was utilized for the knockdown of ZNF143 in U373 cells in subsequent experiments.

### The impact of ZNF143 on the proliferation, migration, and apoptosis of U373 cells

**Figure 5:**
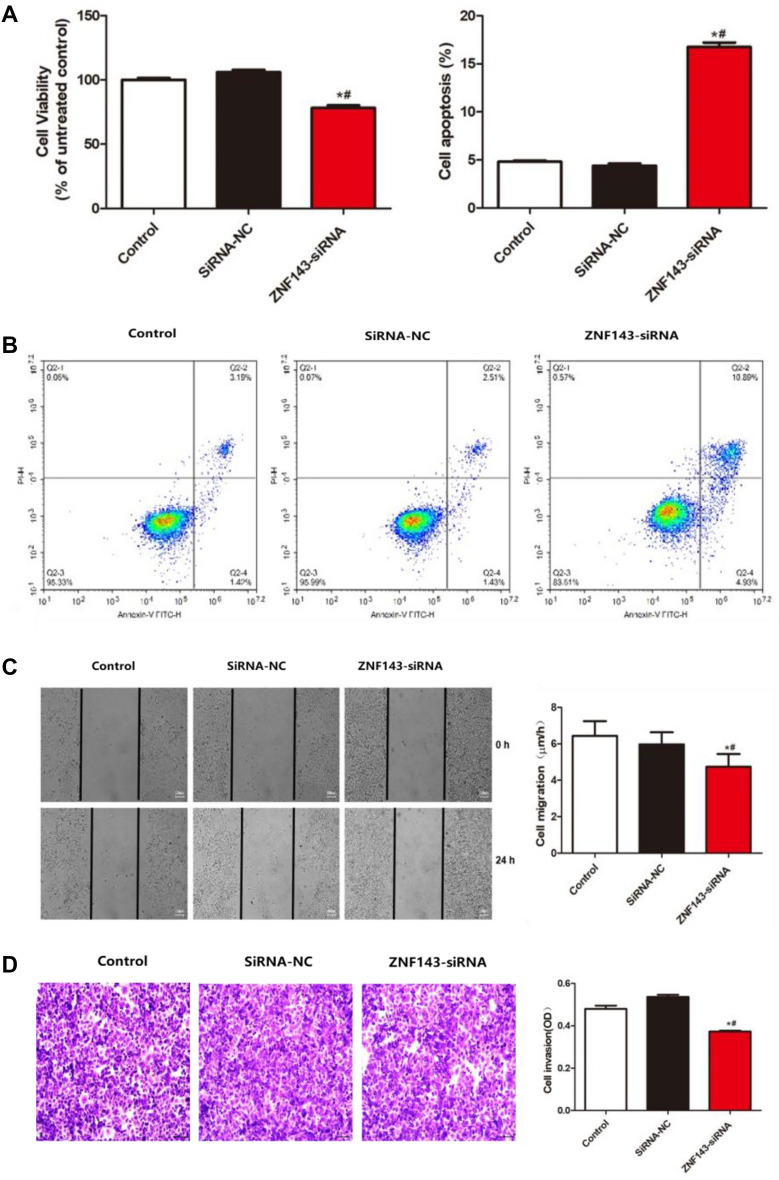
The impact of ZNF143 on the proliferation, migration, and apoptosis of U373 cells. (**A**) Cell viability was detected by CCK-8 assays. (**B**) Apoptosis was evaluated by flow cytometry. (**C**) The wound healing assay was utilized to determine the migration of U373 cells. (**D**) The migration of U373 cells was detected using the Transwell assay (*p < 0.05 vs. the control, ^#^ *p < *0.05 vs. siRNA-NC).

As shown in Fig. 5A, compared to those of the control and siRNA-NC groups, dramatically repressed cell viability was observed in the ZNF143-siRNA group, accompanied by a greatly elevated apoptotic rate (Fig. 5B). Moreover, compared to those of the control and siRNA-NC groups, the value of cell migration (Fig. 5C) and the number of migrated cells (Fig. 5D) were significantly decreased in the U373 cells transfected with ZNF143-siRNA (*p < 0.05 vs. the control, ^#^*p < *0.05 vs. siRNA-NC). These data suggested that the proliferation and migration of U373 cells were inhibited by the knockdown of ZNF143, accompanied by an increased apoptotic rate.

### YAP/TAZ levels were decreased in ZNF143 knockdown U373 cells

**Figure 6:**
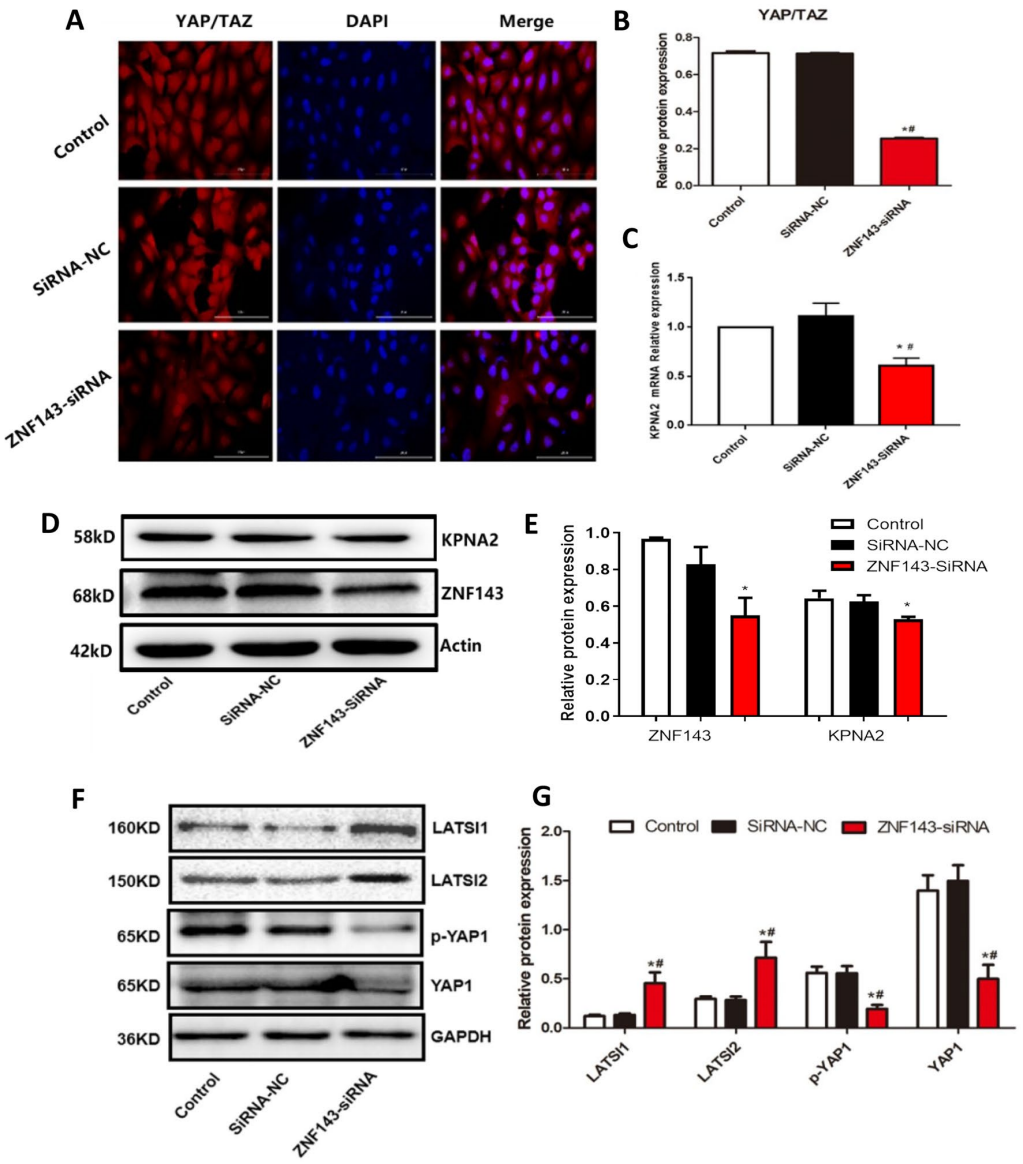
The impact of ZNF143 on the YAP/TAZ pathway. (**A**, **B**) The expression level of YAP/TAZ in U373 cells was determined using the immunofluorescence assay (*p < 0.05 vs. the control, ^#^*p < *0.05 vs. siRNA-NC). (**C**–**E**) The expression level of KPNA2 in the ZNF143 knockdown U373 cells was evaluated by RT‒PCR and Western blotting assays (*p < 0.05 vs. the control, ^#^*p < *0.05 vs. siRNA-NC). (**F**, **G**) The expression levels of LATS1, LATS2, YAP1, and p-YAP1 were determined using Western blotting (*p < 0.05 vs. the control, ^#^*p < *0.05 vs. siRNA-NC).

Subsequently, the level of YAP/TAZ was detected using an immunofluorescence assay in ZNF143 knockdown U373 cells. Compared to those of the control and siRNA-NC groups, the fluorescence intensity (Fig. 6A) was greatly reduced in the U373 cells transfected with ZNF143-siRNA (*p < 0.05 vs. the control, ^#^*p < *0.05 vs. siRNA-NC), suggesting that YAP/TAZ was dramatically downregulated in the ZNF143 knockdown U373 cells.

### KPNA2 levels were positively regulated by ZNF143 in U373 cells

As shown in Fig. 6B,C, compared to those of the control and siRNA-NC groups, KPNA2 was significantly downregulated in the U373 cells transfected with ZNF143-siRNA (*p < 0.05 vs. the control, ^#^*p < *0.05 vs. siRNA-NC), suggesting that the expression level of KPNA2 was positively regulated by ZNF143 in U373 cells.

### The impact of ZNF143 on the expression levels of LATS1, LATS2, YAP1, and p-YAP1 in U373 cells

As shown in Fig. 6D–G, compared to those of the control and siRNA-NC groups, LATS1 and LATS2 were upregulated, while YAP1 and p-YAP1 were downregulated in the U373 cells transfected with ZNF143-siRNA (*p < 0.05 vs. the control, ^#^*p < *0.05 vs. siRNA-NC), suggesting that Hippo signalling in U373 cells was regulated by ZNF143.

## Discussion

KPNA2 is an important member of the Karyopherins α family. Recent studies have shown that KPNA2 is upregulated in a variety of malignant tumours, and the high expression of KPNA2 is closely associated with poor prognosis after surgery and can be used as an independent prognostic indicator in some malignant tumours^8^. Wang found that high expression of KPNA2 was observed in the serum of non-small cell lung cancer (NSCLC) patients, suggesting that KPNA2 could be used as a convenient and fast detection indicator for the postoperative prognosis of tumour patients^9^. The results of the present study showed that KPNA2 was upregulated in glioma cells, indicating that KPNA2 might be involved in the pathogenesis of glioma.

The occurrence and development of malignant tumours are closely related to the proliferation and apoptosis of tumour cells. It has been reported that migration and invasion are repressed in human bladder cancer cells by the knockdown of KPNA2^10^. Furthermore, the proliferation of oesophageal squamous cell carcinoma and endometrial cancer cells was significantly repressed by the silencing of KPNA2 using RNAi technology^7,11^. Proliferation was inhibited and the cell cycle was arrested in G2 phase in U87 MG glioma cells by the knockdown of KPNA2^12^. In the present study, proliferation and migration were significantly decreased, and apoptosis was greatly facilitated in glioma cells by KPNA2 knockdown. These results suggested that the KPNA2 gene played an important role in the growth and metastasis of glioma cells.

The classically activated Hippo pathway plays a role in regulating cell growth, promoting cell damage repair, and inhibiting tumorigenesis by initiating a series of downstream kinase chain reactions^13^. When the Hippo signalling pathway is activated, LATS1⁄2 is phosphorylated and in turn phosphorylates YAP or its homologue TAZ^14^. Phosphorylated YAP/TAZ will remain in the cytoplasm in an inactivated state and will eventually be ubiquitinated and degraded^15^. When the Hippo pathway is blocked, YAP proteins that have not been phosphorylated are overexpressed and accumulate in the nucleus^16^. Abnormal regulation of the Hippo signalling pathway plays an important role in the occurrence and development of tumours, including glioma^17^. Compared with normal brain tissues, the expression levels of LATS1 and LATS2 are significantly decreased in glioma tissues and are associated with poor prognosis^18^. The growth, migration, and invasion of glioblastoma cells are inhibited by the overexpression of LATS1, which regulates cell cycle progression^19^. In glioma cells, NF2 is phosphorylated and inactivated, which upregulates YAP1 expression and mediates the activation of epidermal growth factor receptor and Notch signalling pathways, promoting glioma cell proliferation and tumour formation^20^. The expression level of YAP1 was positively correlated with the grade of glioma, which promotes the proliferation and invasion of glioma cells^21,22^. Similar to YAP1, TAZ expression is elevated in high-grade gliomas. The expression of markers of mesenchymal transformation in glioma stem cells is decreased by the silencing of TAZ, accompanied by the inhibited invasion, self-renewal, and tumour formation of glioma stem cells^23^. Tian et al. showed that high expression of TAZ in gliomas was associated with poor prognosis^24^. The results of the present study showed that LATS1 and LATS2 were upregulated, while YAP1 and p-YAP1 were downregulated in KPNA2 knockdown U373 cells.

Next, we further explored the upstream regulation of KPNA2 and found that ZNF143 might be a specific transcription factor for KPNA2. The transcription factor zinc finger protein 143 (ZNF143) is a human homologue of the Xenopus transcriptional activator Staf and is upregulated in glioma tissues and cell lines^25^. Recent studies have shown that the dysregulation of ZNF143 is closely related to the malignant progression of tumours^26^, which significantly inhibits the migration and metastasis of gastric cancer cells^27,28^. In addition, ZNF143 is dramatically upregulated in lung cancer tissues. High expression of ZNF143 is associated with the high proliferation of lung cancer cells^29^ and poor prognosis of patients^30^. In the present study, a ChIP assay was used to demonstrate that ZNF143 targeted the KPNA2 promoter and positively regulated its expression. Moreover, silencing ZNF143 significantly inhibited the biological behaviour of glioma cells, suggesting that ZNF143 might play an oncogenic role in glioma cells. These results suggested that ZNF143 was involved in the regulation of malignant biological behaviour of glioma cells, and ZNF143 promoted the malignant progression of glioma cells by targeting and positively regulating the expression of the KPNA2 promoter.

In conclusion, the present study revealed that KPNA2 regulates biological behaviours, such as the proliferation, migration, invasion and apoptosis of glioma cells, by regulating the Hippo signalling pathway. Moreover, ZNF143 targeted the promoter region of KPNA2 and positively regulated the expression level of KPNA2, which formed a positive feedback loop to jointly regulate the malignant progression of glioma cells.

## Supplementary Information

Supplementary Figures.

Supplementary Figures.

## Data Availability

The datasets generated and/or analysed during the current study are available in the Chinese Glioma Genome Atlas (CGGA) dataset and Glioma RNA-seq is obtained from TCGA database through UCSC Xena portal, [CGGA: http://www.cgga.org.cn/index.jsp; TCGA: https://xenabrowser.net/datapages/].
