# Supplementary material for: ZNF143 facilitates the growth and migration of glioma cells by regulating KPNA2-mediated Hippo signalling
Source: Sci Rep. 2023 Jul 9;13:11097. doi: 10.1038/s41598-023-38158-x (PMC10330185; doi:10.1038/s41598-023-38158-x)

All the proteins are transferred to the same PVDF membrane, before hybridisation, the PVDF membrane was cropped according to the molecular weight of the proteins to be detected.

Fig 2 A

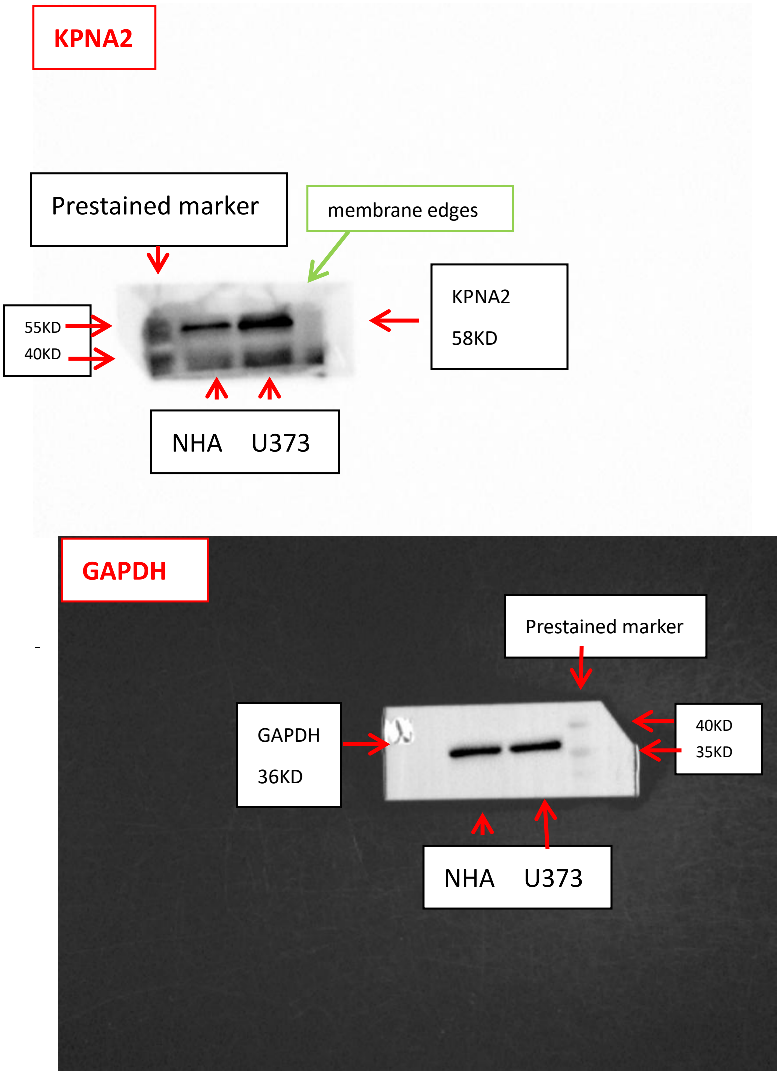

Fig3 C

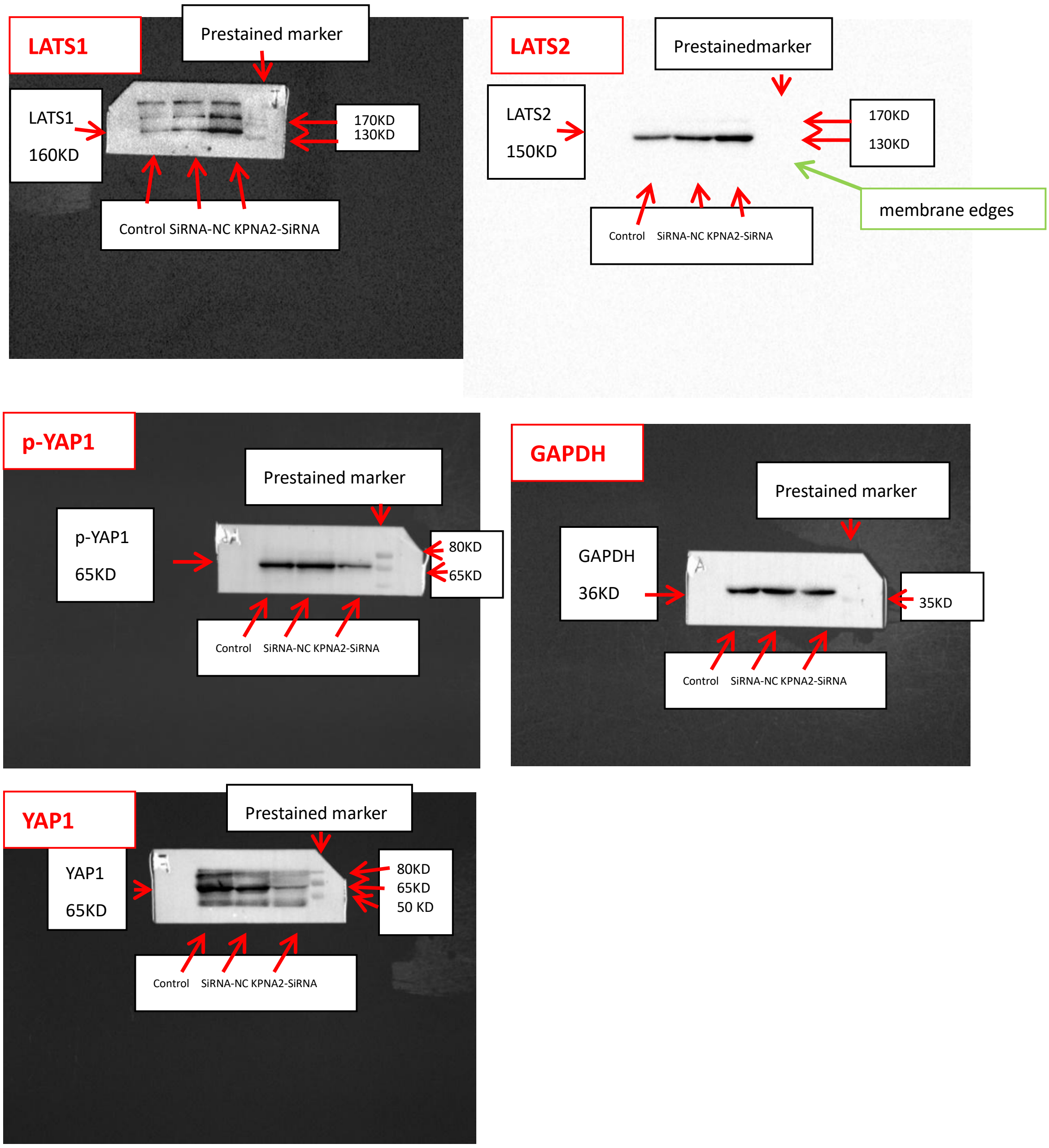

LATS1
Prestained marker
LATS1
160KD
170KD
130KD
Control SiRNA-NC KPNA2-SiRNA
LATS2
Prestainedmarker
LATS2
150KD
170KD
130KD
membrane edges
Control SiRNA-NC KPNA2-SiRNA
p-YAP1
Prestained marker
p-YAP1
65KD
80KD
65KD
Control SiRNA-NC KPNA2-SiRNA
GAPDH
Prestained marker
GAPDH
36KD
35KD
Control SiRNA-NC KPNA2-SiRNA
YAP1
Prestained marker
YAP1
65KD
80KD
65KD
50 KD
Control SiRNA-NC KPNA2-SiRNA

Fig4 A

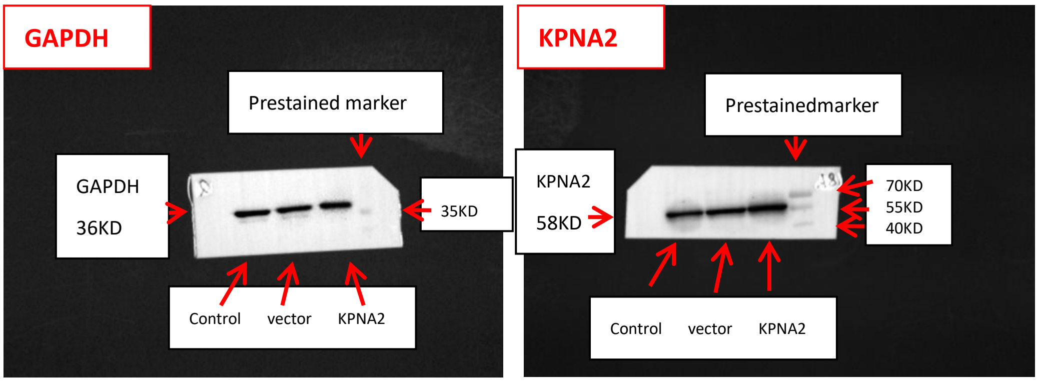
GAPDH
Prestained marker
GAPDH
36KD
35KD
Control
vector
KPNA2
KPNA2
Prestainedmarker
KPNA2
58KD
70KD
55KD
40KD
Control
vector
KPNA2

Fig6 D

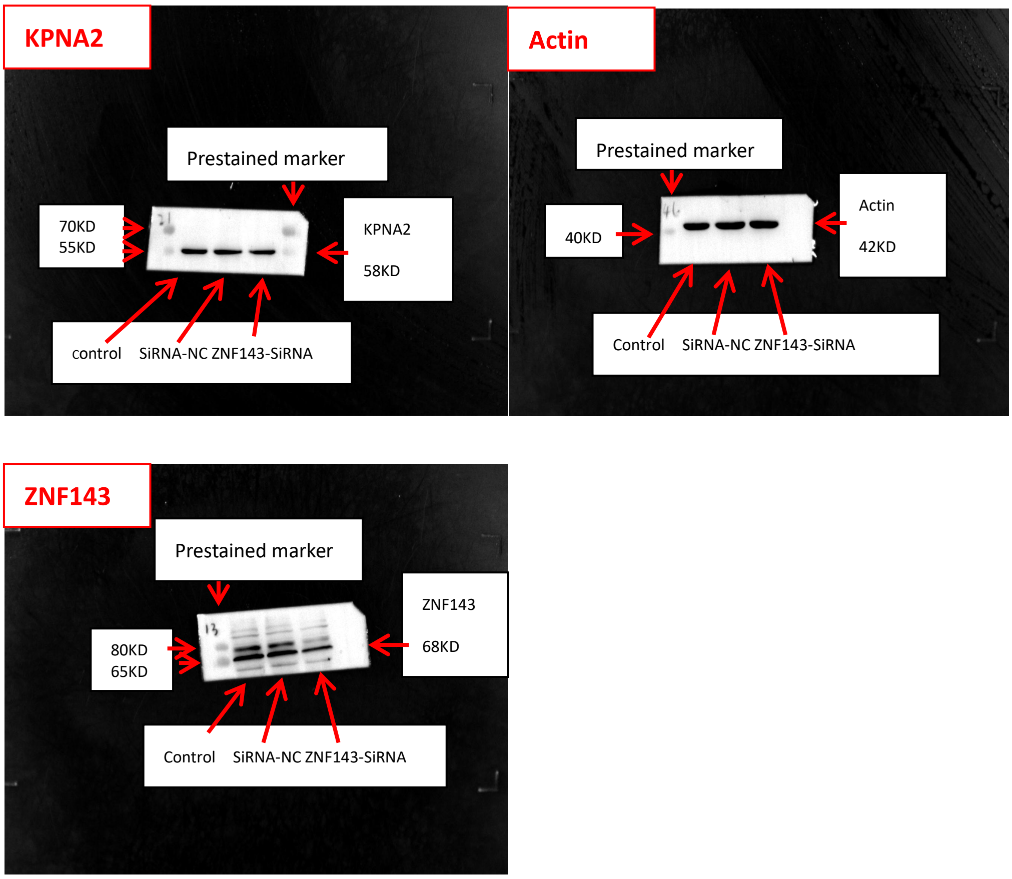
KPNA2
Prestained marker
70KD
55KD
KPNA2
58KD
control SiRNA-NC ZNF143-SiRNA
Actin
Prestained marker
40KD
Actin
42KD
Control SiRNA-NC ZNF143-SiRNA
ZNF143
Prestained marker
80KD
65KD
ZNF143
68KD
Control SiRNA-NC ZNF143-SiRNA

Fig6 F

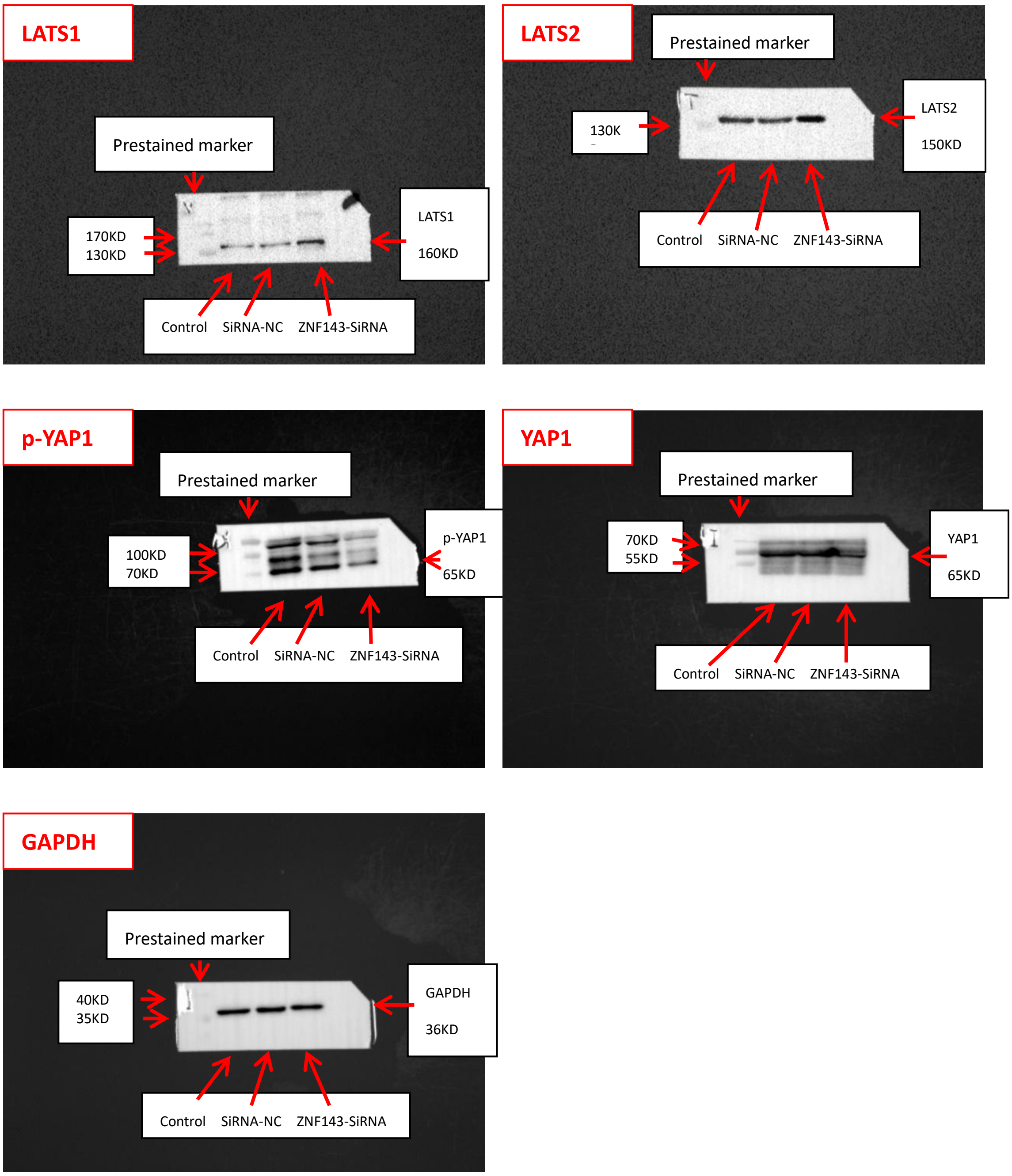
LATS1
Prestained marker
170KD
130KD
LATS1
160KD
Control SiRNA-NC ZNF143-SiRNA
LATS2
Prestained marker
130K
LATS2
150KD
Control SiRNA-NC ZNF143-SiRNA
p-YAP1
Prestained marker
100KD
70KD
p-YAP1
65KD
Control SiRNA-NC ZNF143-SiRNA
YAP1
Prestained marker
70KD
55KD
YAP1
65KD
Control SiRNA-NC ZNF143-SiRNA
GAPDH
Prestained marker
40KD
35KD
GAPDH
36KD
Control SiRNA-NC ZNF143-SiRNA

Supplementary Fig S1

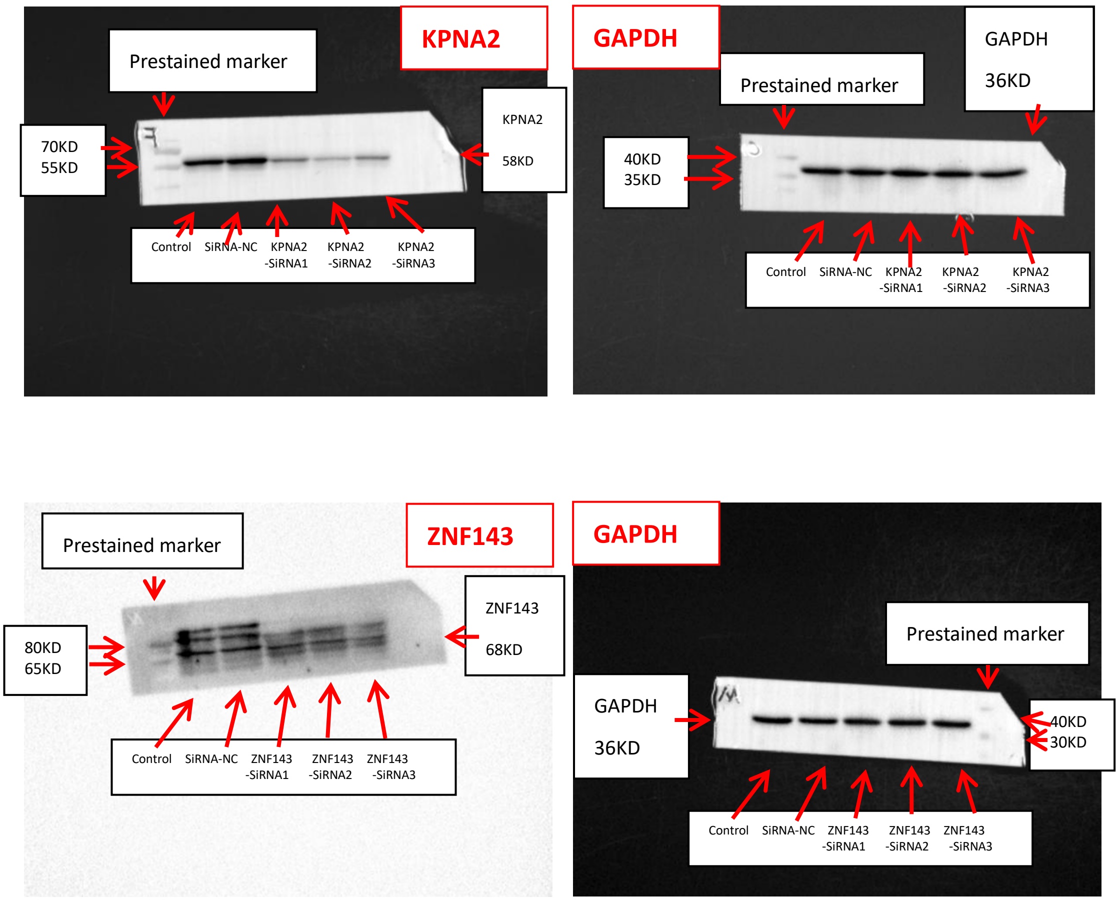

Supplement: Supplementary file 1 — Supplementary Figures. [file 41598_2023_38158_MOESM1_ESM.pdf]
