# Supplementary material for: ZNF143 facilitates the growth and migration of glioma cells by regulating KPNA2-mediated Hippo signalling
Source: Sci Rep. 2023 Jul 9;13:11097. doi: 10.1038/s41598-023-38158-x (PMC10330185; doi:10.1038/s41598-023-38158-x)
Supplement: Supplementary file 2 — Supplementary Figures. [file 41598_2023_38158_MOESM2_ESM.docx]

**Appendix A. Supplementary data**

The following is supplementary data to this article.


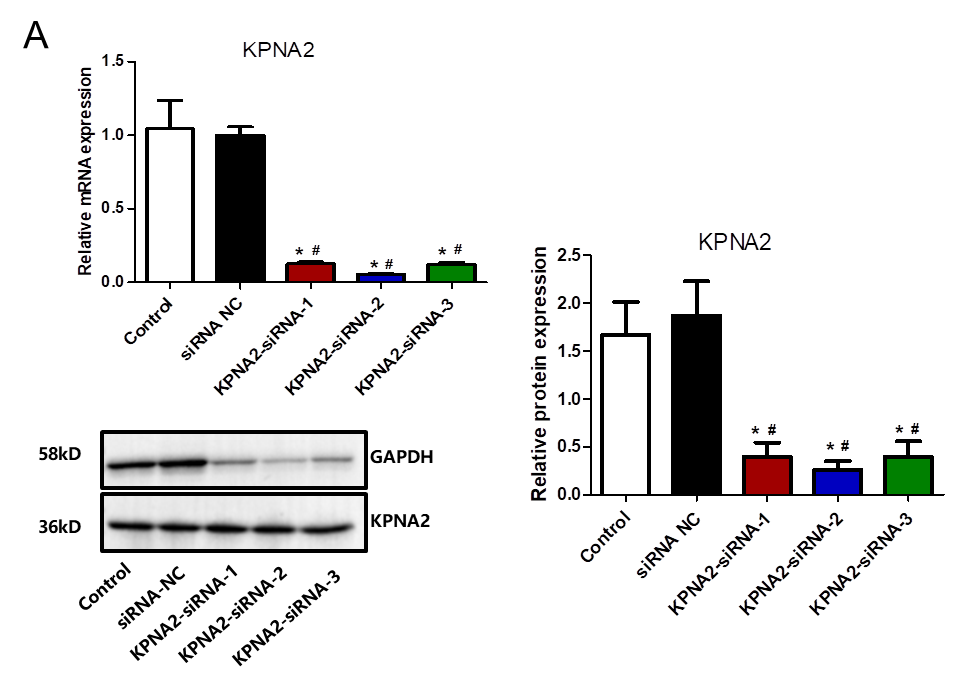


Fig S1. The expression level of KPNA2 in U373 cells transfected with siRNA-NC, KPNA2-siRNA-1, KPNA2-siRNA-2, and KPNA2-siRNA-3 was evaluated by Western blotting and RT-PCR assay (*p<0.05 vs. control, # p<0.05 vs. siRNA-NC).


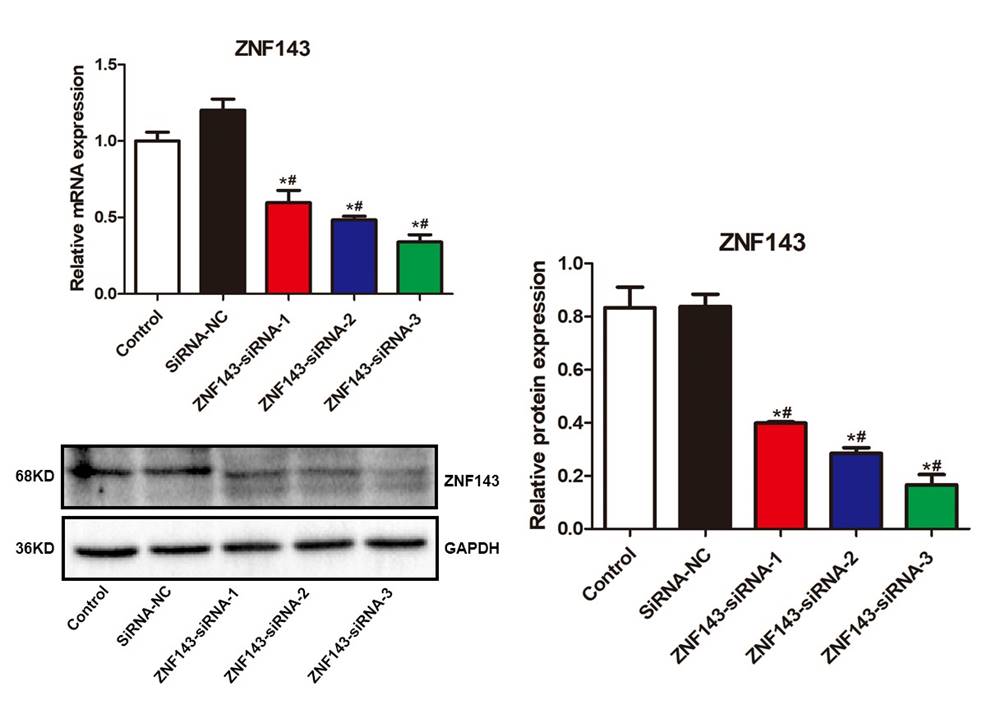


Fig S2. The expression level of ZNF143 in U373 cells transfected with siRNA-NC, ZNF143-siRNA-1, ZNF143-siRNA-2, and ZNF143-siRNA-3 was evaluated by Western blotting and RT-PCR assay (*p<0.05 vs. control, # p<0.05 vs. siRNA-NC).
